# 42.3 METABOLOMICS APPROACHES TO STUDY METABOLIC CO-MORBIDITIES IN PSYCHOTIC DISORDERS

**DOI:** 10.1093/schbul/sby014.176

**Published:** 2018-04-01

**Authors:** Tuulia Hyötyläinen, Tommi Suvitaival, Dawei Geng, Päivi Pöhö, Ismo Mattila, Jaana Suvisaari, Matej Oresic

**Affiliations:** 1 Örebro University; 2 Steno Diabetes Center Copenhagen; 3 University of Helsinki; 4 National Institute for Health and Welfare; 5 University of Turku

## Abstract

**Background:**

Psychotic patients are at high risk for developing obesity, metabolic syndrome and type 2 diabetes. These metabolic co-morbidities are hypothesized to be related to both treatment side-effects as well as to metabolic changes occurring during the psychosis. Earlier metabolomics studies have shown that blood metabolite levels are predictive of insulin resistance and type 2 diabetes in the general population as well as sensitive to the effects of antipsychotics. Here we aimed to identify the metabolite profiles predicting future weight gain and other metabolic abnormalities in psychotic patients.

**Methods:**

We applied metabolomics to investigate serum metabolite profiles in a prospective study setting in 36 first-episode psychosis patients during the first year of the antipsychotic treatment and 19 controls. Two analytical platforms with broad analytical coverage were used. Molecular lipids were analysed by ultra-high performance liquid chromatography coupled to time-of-flight mass spectrometry (UHPLC_QTOFMS) and polar metabolites were analysed by two-dimensional gas chromatography coupled to TOFMS (GCxGC-TOFMS). Ongoing prospective metabolomics studies ae focusing on the subjects in at-risk mental state.

**Results:**

While corroborating several earlier findings when comparing cases and controls and the effects of the antipsychotic medication, we also found that prospective weight gain in psychotic patients was associated with increased levels of triacylglycerols with low carbon number and double-bond count at baseline and independent of obesity, that is, lipids known to be associated with increased liver fat.

**Discussion:**

The first-episode psychotic patients who rapidly gain weight in the follow-up have early metabolic disturbances which are associated with insulin resistance and fatty liver. Our studies suggest that metabolite profiles may be used to identify the psychotic patients most vulnerable to develop metabolic co-morbidities, and may point to a pharmacological approach to counteract the antipsychotic-induced weight gain.

